# Perforator mapping for head and neck reconstructive surgery: a novel personalized approach using magnetic resonance angiography based 3D models and 3D-printing

**DOI:** 10.3389/fonc.2025.1655904

**Published:** 2025-09-30

**Authors:** A.F. de Geer, I. Mulder, L.C. ter Beek, A.S. te Boekhorst, B.I. Plakké, L.H.E. Karssemakers, R. Dirven, P.J.F.M. Lohuis, T.J.M Ruers, F.J. Siepel, P.K. de Koekkoek-Doll, M.J.A. van Alphen, W.H. Schreuder

**Affiliations:** ^1^ Verwelius 3D Lab, Department of Head and Neck Surgery and Oncology, Netherlands Cancer Institute, Antoni van Leeuwenhoek, Amsterdam, Netherlands; ^2^ Robotics and Mechatronics, University of Twente, Enschede, Netherlands; ^3^ Department of Medical Physics, Netherlands Cancer Institute, Antoni van Leeuwenhoek, Amsterdam, Netherlands; ^4^ Department of Radiology, Netherlands Cancer Institute, Antoni van Leeuwenhoek, Amsterdam, Netherlands; ^5^ Department of Otorhinolaryngology and Head and Neck Surgery, Radboud University Medical Center, Nijmegen, Netherlands; ^6^ Department of Surgery, Netherlands Cancer Institute, Antoni van Leeuwenhoek, Amsterdam, Netherlands; ^7^ Faculty of Science and Technology, University of Twente, Enschede, Netherlands; ^8^ Department of Oral and Maxillofacial Surgery, Amsterdam Medical University Center and Academic Center for Dentistry Amsterdam (ACTA), Amsterdam, Netherlands

**Keywords:** magnetic resonance angiography, computer-aided design -computer-assisted manufacturing, head and neck cancer, perforator mapping, reconstructive head and neck surgery, virtual surgical planning (VSP), 3D printing

## Abstract

**Background:**

Reconstruction of large head and neck defects in oncologic patients often requires free vascularized tissue flaps. Successful flap design and elevation depend on accurate preoperative identification of perforator vessels. Preoperative Magnetic Resonance Angiography (MRA) could offer detailed insights into perforator course, caliber, origin, and main pedicle length, and thus is expected to surpass conventional handheld Doppler. This study introduces a novel approach for perforator mapping in reconstructive head and neck surgery that integrates MRA with 3D modelling and 3D-printing.

**Methods:**

The proposed workflow comprises four steps: 1) acquisition of contrast-enhanced MRA, 2) construction of a 3D anatomical model, 3) design and 3D-printing of a patient-specific perforator guide, and 4) transfer of perforator locations from the model to the patient’s skin using the guide. To illustrate the clinical feasibility and potential utility of this approach, an initial cohort of patients undergoing perforator flap surgery for oncologic head and neck reconstruction was included. Flap types included fibula free flap (FFF), anterolateral thigh flap (ALT), and medial sural artery perforator flap (MSAP). Intraoperative findings were compared with the 3D models, and surgeons evaluated the models’ usability for virtual planning of flap design and elevation using a five-point Likert scale questionnaire.

**Results:**

Ten patients were included for analysis: three FFF, two ALT, and five MSAP cases. In FFF and ALT patients, all perforators intraoperatively used for flap elevation were successfully visualized on MRA and represented in the 3D models. In MSAP patients, small-caliber perforators were not consistently visible. The mean absolute difference between pedicle lengths measured in the 3D models and intraoperatively was 1.0 cm (SD 0.9 cm). The usability questionnaire yielded an average score of 4.2 out of 5, suggesting the potential of MRA-based 3D models for virtual surgical flap planning.

**Conclusions:**

This is the first study to combine preoperative MRA with 3D modelling and 3D-printing for perforator mapping in head and neck reconstruction. The workflow offers a radiation-free, patient-specific planning tool that may enhance surgical precision and support personalized flap design in complex oncological cases.

## 1 Introduction

Surgical treatment of patients with advanced head and neck cancer often leads to large and complex defects, requiring reconstructive surgical procedures. These tissue defects can compromise critical functions such as speech, mastication, and swallowing, as well as facial aesthetics. Free tissue transfer using microsurgical techniques has become the standard of care for addressing these challenges (1–5).

The selection of a donor site for flap harvest is guided by the specific characteristics of the defect and the type of tissue required for reconstruction. For bony reconstruction of the craniomaxillofacial skeleton, the free fibula flap (FFF) is the main workhorse flap, while the anterolateral thigh flap (ALT) and free radial forearm flap (FRFF) are most commonly used for reconstruction of soft tissue defects (2, 6). With the growing preference for perforator flaps, owing to their reduced donor site morbidity and enhanced versatility in flap design, the medial sural artery perforator (MSAP) flap has emerged as promising alternative to the FRFF (7–11).

Accurate identification and meticulous dissection of perforator vessels are essential for designing, elevating, and ensuring the survival of the flap (2). However, the location and vascular territory of perforators can vary significantly between patients. Several imaging modalities are available for preoperative perforator mapping, with Computed Tomography Angiography (CTA) considered the gold standard (12, 13). Despite this, handheld Doppler remains the most used tool in clinical practice even though it has a high rate of false positives and false negatives (12–15). Moreover, handheld Doppler does not provide information about the inter- or intramuscular course of the perforators or the length of the main pedicle that can be achieved. This information is essential, especially in more complex head and neck reconstructions that require chimeric flaps, multiple skin paddles or a long vascular pedicle in vessel-depleted necks (16). While CTA provides these valuable anatomical details, it exposes patients to ionizing radiation and nephrotoxic contrast agents (12, 17). In contrast, Magnetic Resonance Angiography (MRA) provides a radiation-free alternative with superior soft tissue contrast, potentially enhancing visualization of the perforator course in relation to surrounding musculature (18–21).

In bony craniomaxillofacial reconstruction, the integration of virtual surgical planning (VSP) and computer-assisted design and manufacturing (CAD-CAM) has become standard practice in many centers, offering significant clinical benefits (22–24). However, the application of these technologies to soft tissue reconstruction is limited, primarily due to complex challenges that have yet to be overcome. Current intraoperative techniques for flap modelling and shaping heavily rely on the surgeon’s experience and preference, often lacking a personalized approach. Typically, a large flap is harvested through a wide incision and subsequently resized during inset, frequently necessitating skin grafting at the donor site, thereby increasing morbidity (25).

Three-dimensional (3D) visualization of soft tissues of both donor- and recipient site could offer several advantages. These include patient-specific modelling of complex defects, improved estimation of flap dimensions and volume, and enhanced understanding of the intricate vascular anatomy of perforators. Potentially, this could lead to more efficient surgery reducing operating times and donor site morbidity, while improving functional outcomes for the patient (25–28).

An important first step towards 3D VSP for soft tissue reconstruction in complex head and neck surgery is to create a personalized virtual model of the macro- and microvascular anatomy, along with accurate intraoperative translation. In this study, we present a novel MRA-based workflow for generating detailed 3D vascular models, enabling precise mapping of perforators and pedicle anatomy while visualizing relevant surrounding structures. The primary aim of this work was to develop a reliable and robust MRA-based virtual 3D model of the vascular anatomy and to evaluate the potential usability of this model for preoperative planning of flap harvesting, thereby establishing a foundation for personalized and reproducible soft tissue planning for head and neck reconstruction.

## 2 Materials and methods

A prospective observational study was conducted involving an initial patient cohort. Between April 2023 and August 2024, all consecutive patients scheduled for head and neck tumor resection with ALT, MSAP or FFF reconstruction at the Antoni van Leeuwenhoek Hospital were eligible for inclusion. Included patients underwent preoperative MRA imaging of the donor site according to the current clinical protocol, which was adapted during the course of the study based on emerging insights. Exclusion criteria included general contraindications to MRI, such as non-MRI compatible implants, pacemakers, claustrophobia and allergies for gadolinium-based contrast agents. Written informed consent was obtained from all patients prior to inclusion.

### 2.1 Workflow

#### 2.1.1 Overview

**Figure 1:**
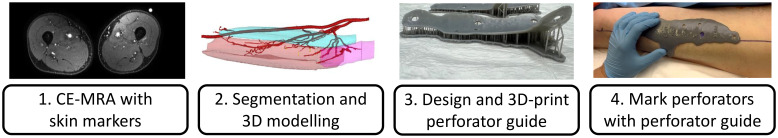
Overview of proposed MRA-based perforator mapping workflow with different steps. CE-MRA, contrast-enhanced Magnetic Resonance Angiography.

Preoperatively, a contrast-enhanced MRA (CE-MRA) is acquired with skin markers on the donor site of interest (step 1). The MRA is converted into 3D models comprising the perforators, arterial branches of the main vascular pedicle and relevant surrounding anatomy including muscles, septa, fascia, bones, and skin (step 2). Based on the 3D model of the skin, skin markers and perforator locations, a patient-specific perforator guide is designed and 3D-printed (step 3). The perforator guide is used to translate the perforator locations of the 3D model to the patient in the operating room. Handheld Doppler is used to verify an arterial signal at the locations marked with the perforator guide (step 4). See **Figure 1** for an overview of the workflow. In the sections below, the workflow is described step-by-step with practical recommendations.

#### 2.1.2 Image acquisition

CE-MRA images of the donor site are acquired with a 3.0 T Philips Achieva dStream scanner (Philips Healthcare, Best, the Netherlands) using a 32-channel Torso coil. Before image acquisition, three to five cod liver oil spheres are placed on birthmarks or, if absent, on pen marked points within the donor area of interest to serve as MRI fiducial markers for relating the patient’s anatomy in a coordinate system.

For the three donor sites, different patient scanning positions are used to resemble the intraoperative positioning. Patients scheduled for ALT reconstruction are positioned in supine position with stretched legs. Patients scheduled for FFF reconstruction are positioned in the supine position with the knee in a flexed position to ensure that the muscles in the leg are not compressed, thereby maximizing the flow of perforators in the calf. Patients scheduled for MSAP reconstruction are positioned in prone position to maximize flow of perforators in the calf and to mimic the position that is used in our center for preoperative perforator mapping with handheld Doppler. For all cases, the ankle and foot are stabilized with sandbags to make sure the patient stays in position for the duration of the scan.

**Figure 2:**
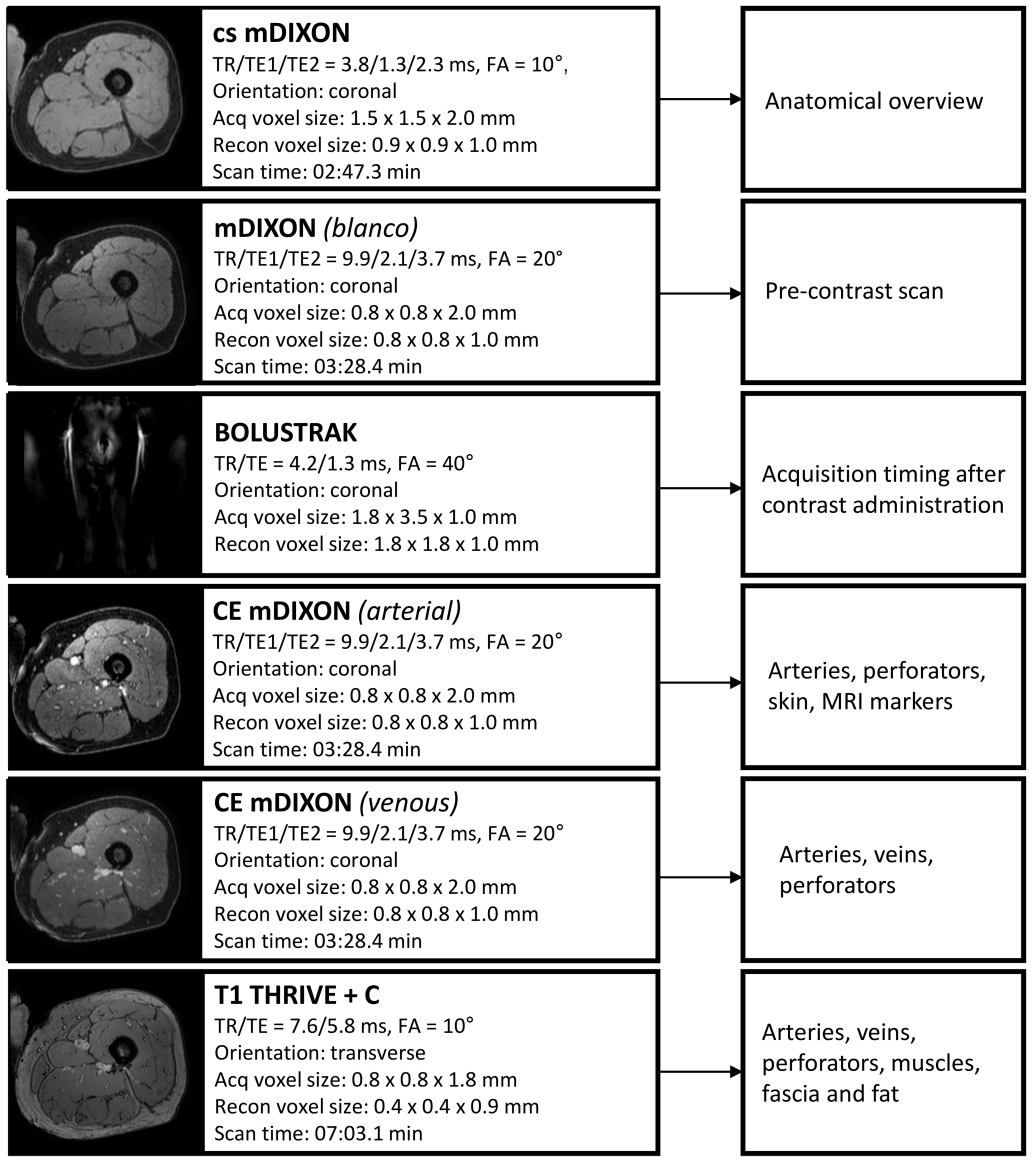
Overview of Magnetic Resonance Angiography protocol with scan parameters. TR, repetition time; TE, echo time; Acq, acquisition; recon, reconstruction; CE, contrast-enhanced; FA, flip angle.

The scan duration of the MRI protocol is 20 minutes. The protocol comprises of three T1-weighted 3D gradient echo sequences with parameters summarized in **Figure 2**. The protocol starts with a non-contrast-enhanced mDIXON to create an overview of the donor site anatomy and to be used for planning the field of view for the remaining sequences. Next, a three-phase contrast-enhanced mDIXON is acquired consisting of a pre-contrast scan, contrast injection with fluoroscopic triggering (2D BOLUSTRAK), an arterial scan and a venous scan. For the contrast-enhanced scans, 15 ml of a gadolinium-based contrast agent (Clariscan™ 0.5 mmol/ml, GE Healthcare AS, Oslo, Norway) is injected at a rate of 5 ml/s, followed by 30 ml saline flush at the same rate. Finally, a THRIVE sequence is used as late venous phase scan.

**Figure 3:**
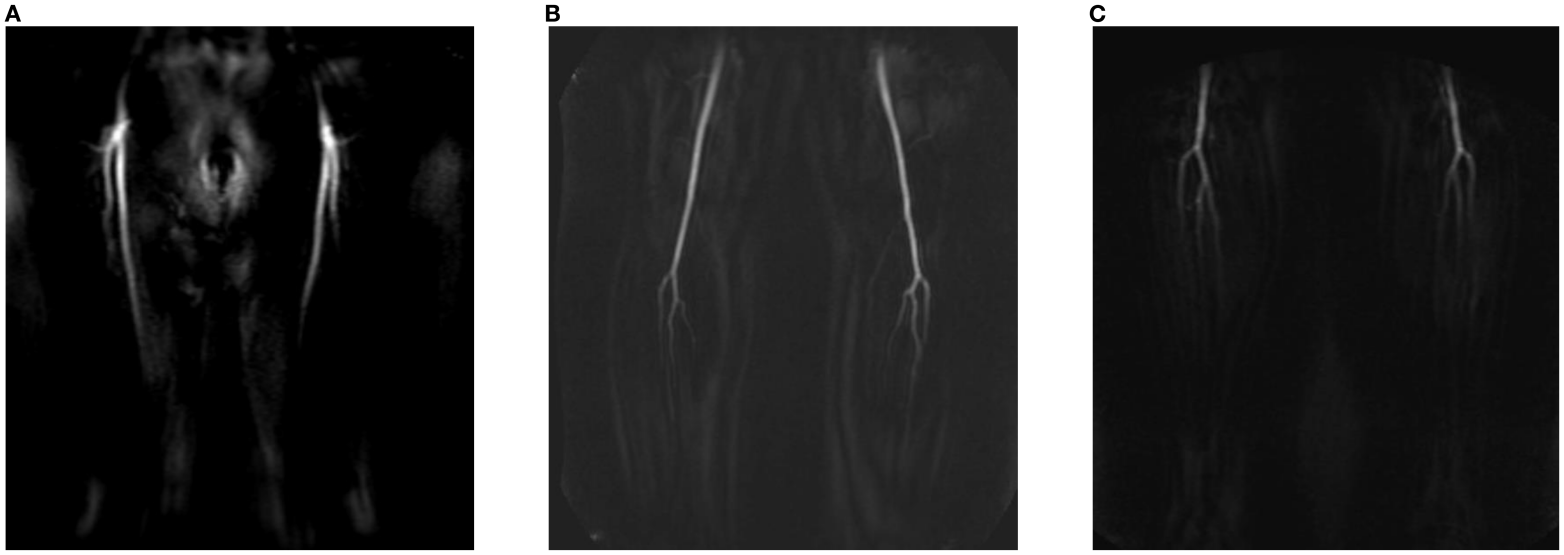
View of BOLUSTRAK image when dynamic scan is started for **(A)** anterolateral thigh flap, **(B)** medial sural artery perforator flap, **(C)** fibula free flap.

**Figure 4:**
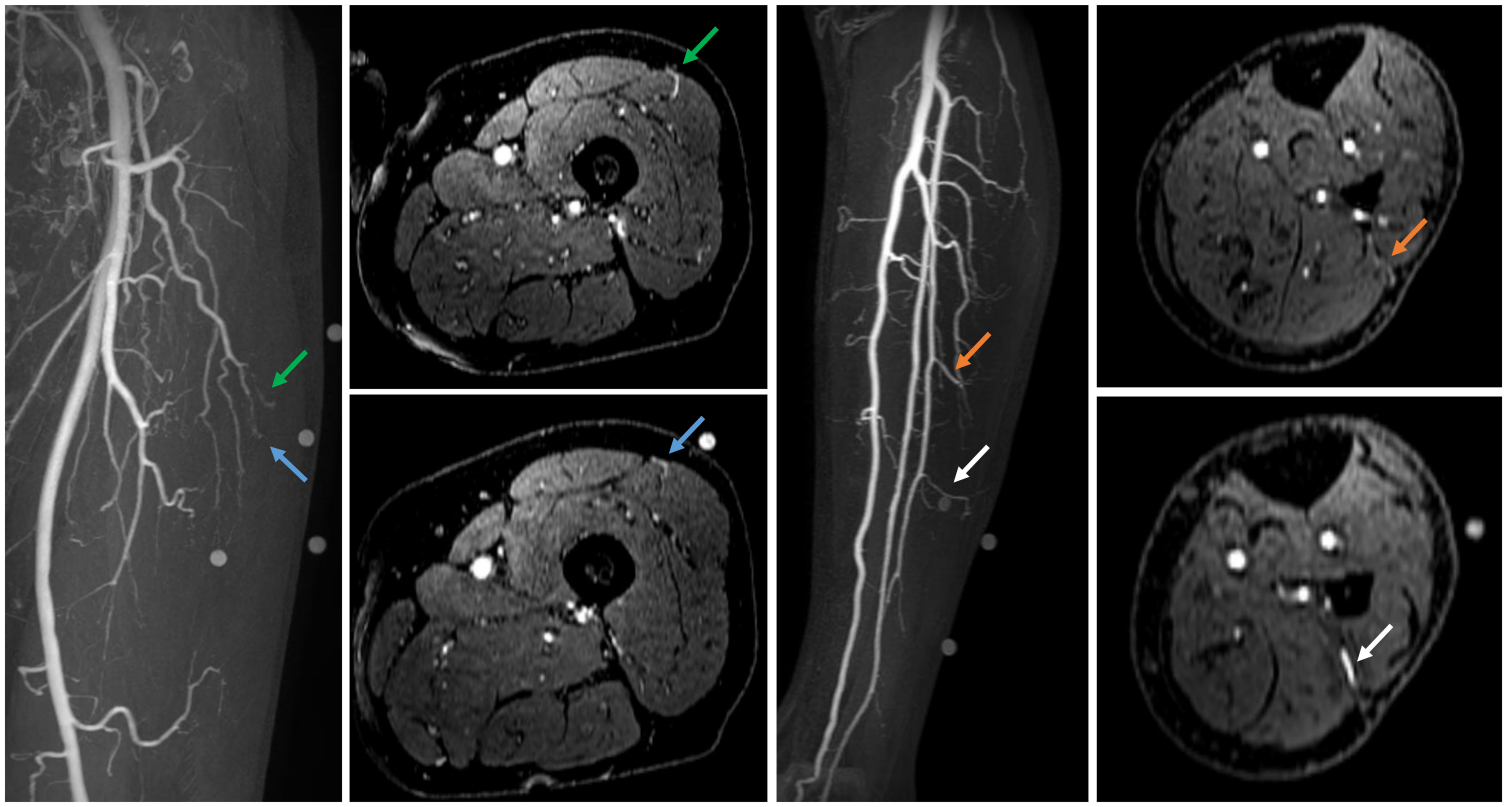
Contrast-enhanced mDIXON arterial Maximum Intensity Projection (MIP) images with corresponding axial slices, in which perforators are indicated by arrows. The left panel shows the upper leg for the anterolateral thigh flap, while the right panel depicts the lower leg for the fibula free flap.

Centra k-space acquisition is used for contrast filling, starting two seconds after fluoroscopic triggering. For optimal perforator filling in the head and neck cancer patient population, the arterial mDIXON scan is triggered when vascular images are seen as shown in **Figure 3**. For ALT patients, branching of the lateral circumflex femoral artery (LCFA) is visible and contrast fills half of the upper leg. For MSAP and FFF patients, the popliteal trifurcation is visible and contrast fills the upper third of the lower leg. **Figure 4** displays examples of arterial mDIXON Maximum Intensity Projection (MIP) images alongside corresponding axial slices, illustrating visible perforators in patients scheduled for surgery with ALT and FFF reconstruction.

#### 2.1.3 Segmentation and 3D modelling

The Digital Imaging and Communications in Medicine (DICOM) data of the MRA protocol are exported and loaded into Materialise Mimics software (version 24 or newer, Materialise, Leuven, Belgium) for segmentation and 3D modelling. The arterial mDIXON is used for perforator identification and segmentation and therefore chosen as reference scan. The other scans are registered to this reference scan with the automatic registration module. Segmentations are performed semi-automatically by thresholding with “Edit mask” tool and “Multiple Slice Edit” tool and optimized with morphology operations. When finished, the segmentations are converted into 3D models and are visualized as overlay on coronal, sagittal, and axial MRA slices for visual inspection of segmentation accuracy. The 3D models can be viewed on a screen and rotated for inspection at different angles.

#### 2.1.4 Design and 3D-printing of perforator guide

Since the lower extremities lack clear and reproducible anatomical landmarks for creating a coordinate system to translate the perforator locations of the MRA-based 3D model to the patient in the operating room, a patient-specific perforator guide is 3D-printed. The perforator guide is designed based on the patient’s skin segmentation and the MRI markers. The guide is designed in Materialise 3-matic software (version 16 or newer, Materialise, Leuven, Belgium) by marking the area of interest on the skin segmentation model and creating a base with 3.0 mm thickness. Cylindrical holes with radius of respectively 5.0 mm and 3.0 mm are added on the locations of the MRI markers and points where the perforators penetrate the deep fascia. The perforator guide is saved in Standard Tesselation Language (STL) and loaded into PreForm software (V3.7 or newer, Formlabs Inc., MA, USA). The guide is 3D-printed using skin-friendly Tough 1500 V1 resin (Formlabs Inc., MA, USA) using a stereo lithography Form 3BL printer (Formlabs Inc., MA, USA) with layer thickness 0.1 mm and default printer settings. To ensure smooth fit of the guide on the patient’s skin, the guide is positioned in such orientation that supports are added at the outer side of the guide. Support settings with touchpoint density 80% and touchpoint size 0.50 mm are used. Post-processing is performed following the manufacturer’s guidelines: washing for 20 minutes in isopropanol using a Form Wash device (Formlabs Inc., MA, USA) and curing for 60 minutes at 60°C using a BB Cure XL photo-curing device (Meccatronicore, Trente, Italy).

#### 2.1.5 Translation of perforator model to operating room

Marking of perforator locations on the patient’s skin with the 3D-printed perforator guide is performed before surgery or in the operating room just before incision, up to the surgeon’s preference. The perforator guide is positioned on the donor site with help of the MRI marker locations in the guide and the perforator locations are marked on the skin with a surgical pen. After removing the guide, the marked perforator locations can be verified with handheld Doppler (Huntleigh Dopplex DMX, Cardiff, UK).

### 2.2 Evaluation

#### 2.2.1 MRA imaging and 3D model

The initial patient cohort described in this study was used to assess the feasibility of step 1 and 2 of the proposed workflow. All patients underwent surgery following the standard hospital protocol, including perforator mapping using handheld Doppler. Additionally, the operating surgeons (LK, WS) were granted preoperative access to the 2D MRA images. However, the 3D models were intentionally withheld until after surgery to evaluate their concordance with intraoperative findings without influencing clinical decision making.

During surgery, the location, origin, and course of perforators involved in flap elevation were documented. The *in-situ* pedicle length, defined as the distance from the intended ligation point to the site where the perforator penetrates the deep fascia en route to the skin, was measured intraoperatively with a surgical ruler.

Postoperatively, the intraoperative observations were compared with both the 2D MRA images and the 3D models. Digital measurements of pedicle length in the 3D models were performed by the researcher (AG). Furthermore, the 3D models were presented to the operating surgeons, who were then asked to complete a questionnaire consisting of seven statements assessing the usability of the MRA-based 3D models for preoperative virtual surgical flap planning. Responses were recorded using a five-point Likert scale.

This prospective observational study was performed according to the guidelines of the Declaration of Helsinki and ethical approval was obtained from the Institutional Review Board of the Netherlands Cancer Institute – Antoni van Leeuwenhoek (IRBdm22-042). The initial four included patients were excluded from analysis as they were used to familiarize the team with MRA image interpretation and the 3D modelling workflow. Descriptive statistics (mean, median, standard deviation, range) were used for interpretation of the results.

#### 2.2.2 3D-printed perforator guide

During patient inclusion, the concept of a 3D-printed perforator guide was created to transfer perforator locations from the 3D model to the patient’s anatomy during surgery (steps 3 and 4 of the proposed workflow). The feasibility of this approach is demonstrated through a case illustration.

## 3 Results

### 3.1 Patient characteristics

Seventeen patients scheduled for surgery with eighteen perforator flaps were included in this study with a mean age of 67 years (range 40-90 years). The majority of the patients were scheduled for FFF reconstruction (44%). Patient demographics are shown in **Table 1**.

**Table 1:** Demographics of included patients.

Sex	Number (percentage)
Male	10 (58.8%)
Female	7 (41.2%)
Median age, years (range)	70 (40–90)
Surgical indication	
OSCC	13 (76.5%)
SCC	1 (5.9%)
ACC	1 (5.9%)
Skin melanoma	1 (5.9%)
Secondary maxillary reconstruction	1 (5.9%)
Defect site	
Left	6 (35.3%)
Right	9 (52.9%)
Both	2 (11.8%)
Scheduled flap type	
FFF	8 (44.4%)
ALT	5 (27.8%)
MSAP	5 (27.8%)

OSCC, oral squamous cell carcinoma; CSCC, cutaneous squamous cell carcinoma; ACC, adenoid cystic carcinoma; FFF, fibula free flap; ALT, anterolateral thigh flap; MSAP, medial sural artery perforator flap.

**Figure 5:**
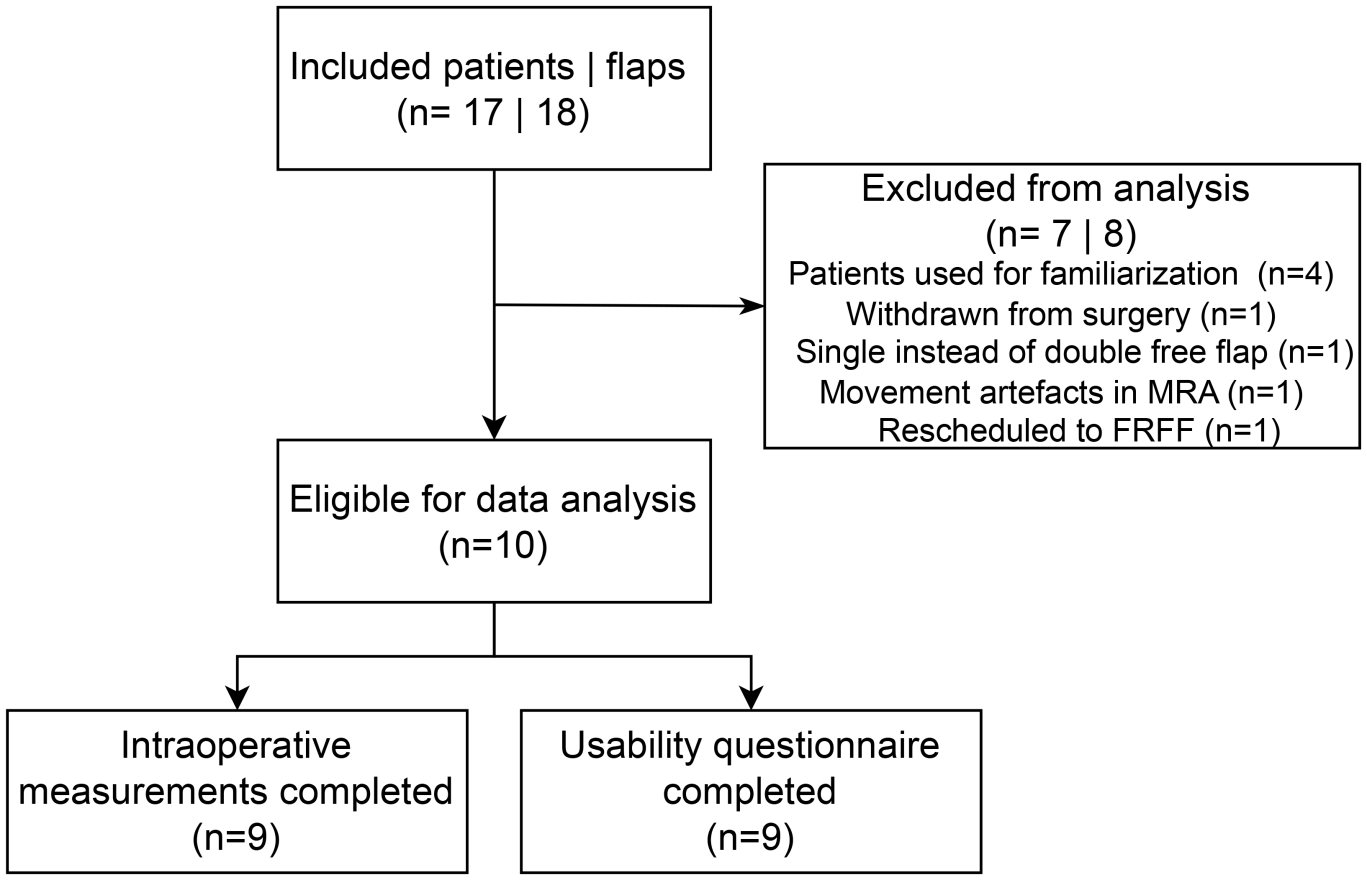
Consort diagram of included patients. MRA, magnetic resonance angiography; FRFF, free radial forearm flap.

Additionally, beyond the scans of the four patients involved in workflow familiarization, four other scans could not be analyzed. One patient withdrew from surgery. Another patient was scheduled for double free flap surgery involving both the fibula free flap (FFF) and the anterolateral thigh (ALT) flap; however, only the FFF was harvested. Another patient was excluded due to movement artefacts in MRA imaging, which limited perforator visualization. Additionally, one patient underwent surgery using a radial forearm flap instead of an ALT flap due to the presence of atherosclerotic vessels in the leg, as identified on MRA imaging. Consequently, a total of ten patients, each receiving a single flap, were included for final analysis (see **Figure 5**).

### 3.2 MRA imaging and 3D model

**Figure 6:**
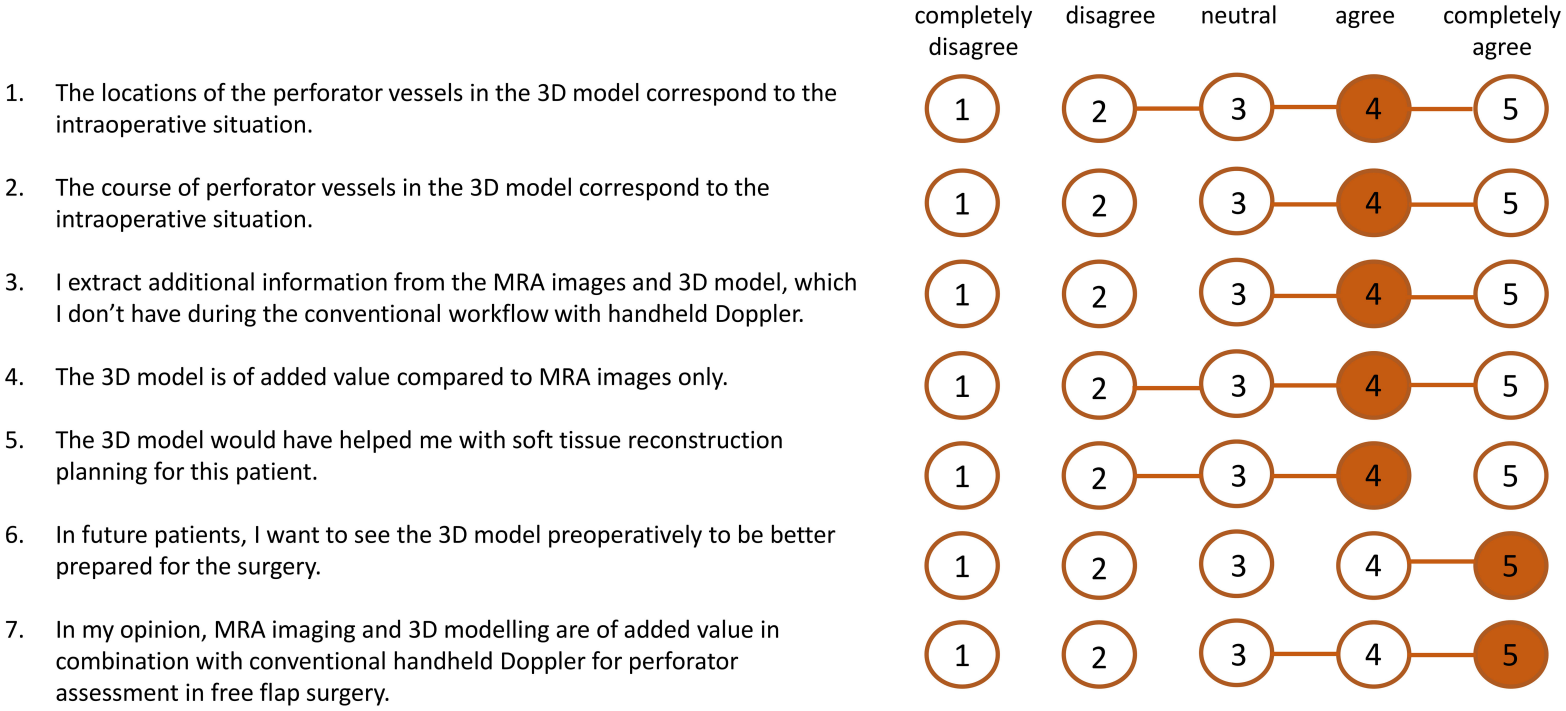
Results of scores of usability questionnaire (n=9 patients). The orange numbers indicate the median score and the horizontal orange lines show the range of scores.

**Figure 7:**
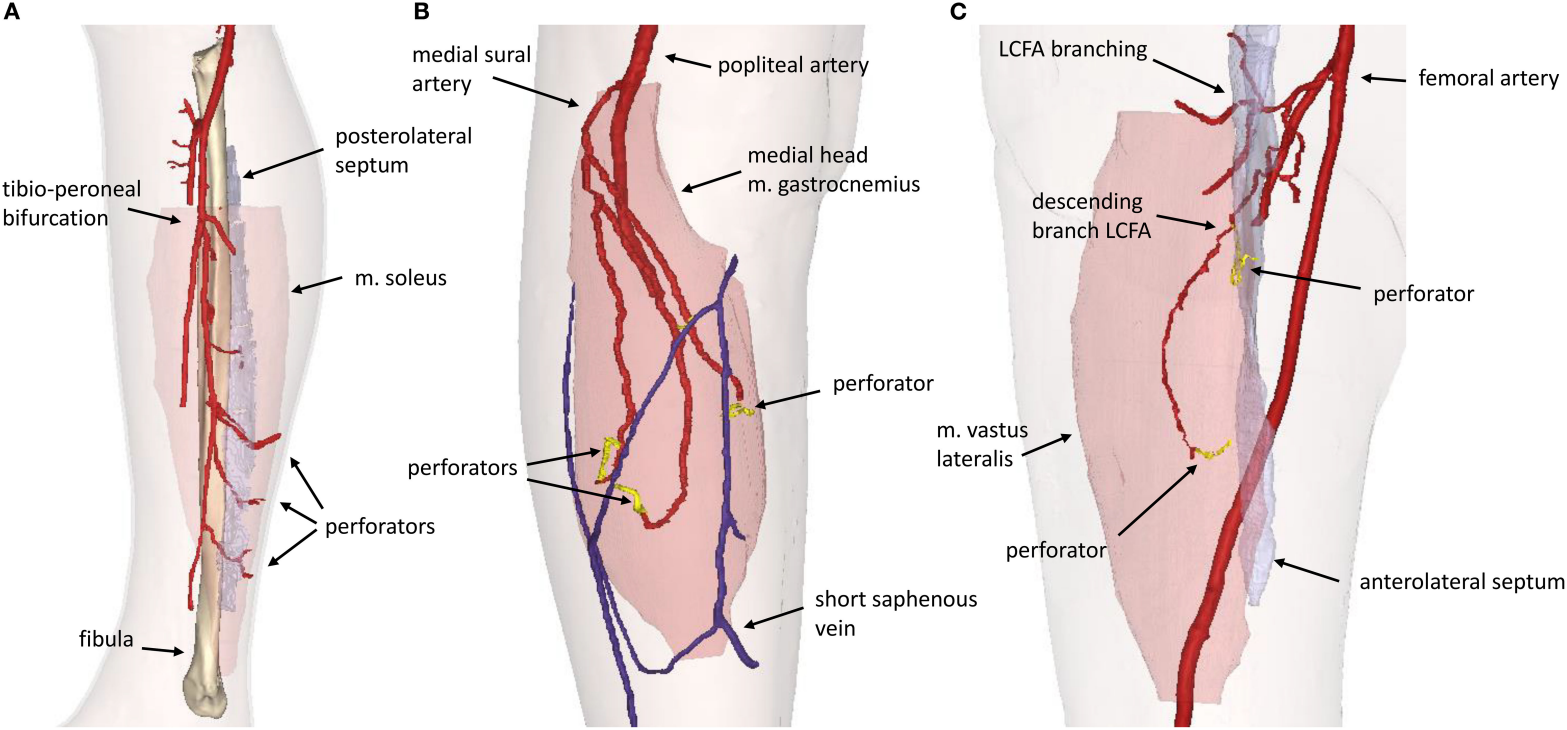
3D model visualization for **(A)** Fibula Free Flap, **(B)** Medial Sural Artery Perforator Flap, **(C)** Anterolateral Thigh Flap. LCFA, lateral circumflex femoral artery.

The mean absolute difference (SD) in pedicle length measured in the 3D model and intraoperatively was 1.0 (0.9) cm (**Table 2**). The mean (SD) score for the usability questionnaire was 4.2 (0.6) out of 5.0 points indicating the usability of the 3D models for preoperative virtual planning of flap design and flap elevation (**Figure 6**). A visualization of a 3D model for a FFF, MSAP, and ALT patient is shown in **Figure 7**.

**Table 2:** Comparison of intraoperatively identified perforators included in flap elevation with those visualized on contrast-enhanced mDIXON images and integrated into the 3D models.

ID	Donor site	Pedicle length (cm)			Perforator visible in CE-mDIXON images (Yes/No)	Perforator incorporated in 3D model (Yes/No)	Perforator course matched with imaging findings (Yes/No)
		Intra- operative	3D model	Difference			
**1**	FFF	9.5	9.5	0.0	1. Yes	1. Yes	1. Yes
**2**	MSAP	2.5*	3.0*	-0.5	**1. Yes** 2. No	**1. Yes** 2. No	**1. Yes** 2. n.a.
**3**	ALT	9.0	7.1	1.9	1. Yes	1. Yes	1. Yes
**4**	FFF	10.5	9.9	0.6	1. Yes	1. Yes	1. Yes
**5**	MSAP	15.3	12.4	2.9	1. No 2. No **3. Yes**	1. No 2. No **3. Yes**	1. n.a. 2. n.a. **3. Yes**
**6**	MSAP	9.0	9.3	-0.3	**1. No** 2. No	**1. Yes** 2. Yes	**1. Yes** 2. Yes
**7**	MSAP	15.3	15.9	-0.6	1. No **2. Yes**	1. No **2. Yes**	1. n.a. **2. Yes**
**8**	FFF	11.5	10.0	1.5	**1. Yes** 2. Yes 3. Yes	**1. Yes** 2. Yes 3. Yes	**1. Yes** 2. Yes 3. Yes
**9**	ALT	14.0	13.7	0.3	**1. Yes** 2. Yes	**1. Yes** 2. Yes	**1. Yes** 2. Yes
**10**	MSAP	Not measured	n.a.	n.a.	1. No	1. No	1. n.a.

Perforators used for pedicle length measurements are highlighted in bold. ALT, anterolateral thigh flap, MSAP, medial sural artery perforator flap; FFF, fibula free flap. CE, contrast-enhanced; n.a., not applicable.

* Length measured at level of MSA sub-branching, because the full length could not be assessed in the 3D model, as pedicle ligation was performed at a random location that could not be identified within the model.

#### 3.2.1 FFF and ALT patients

In patients receiving ALT or FFF reconstruction (n=5), 100% of perforators intraoperatively used for flap elevation were visible in CE-mDIXON images and incorporated in the 3D model. In addition, the inter- or intramuscular course matched for all perforators (**Table 2**).

#### 3.2.2 MSAP patients

**Figure 8:**
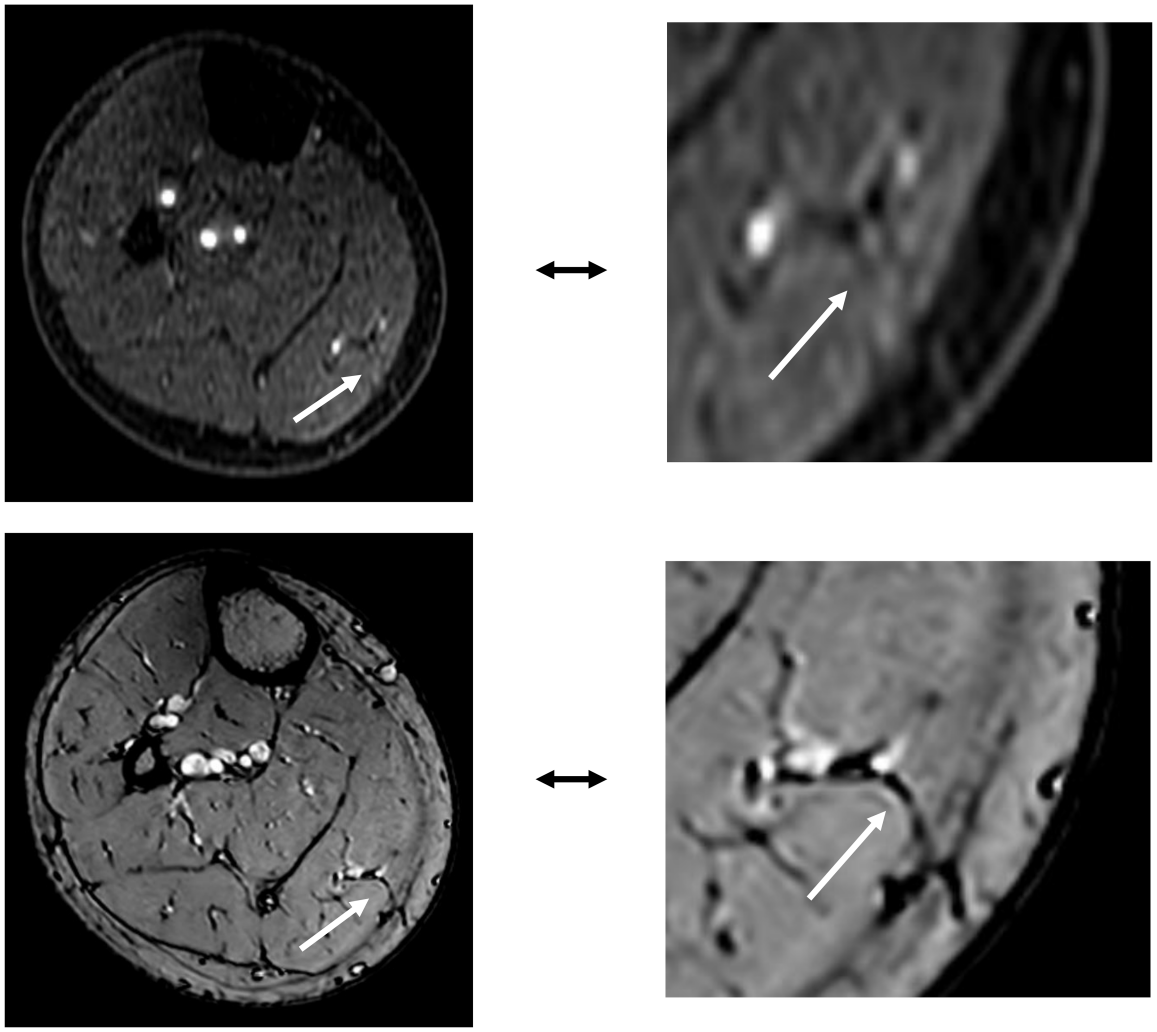
The top image shows an MSAP perforator as visualized on a contrast-enhanced mDIXON image. The bottom image depicts the same MSAP perforator as a fat plane penetrating the deep fascia, identified on a THRIVE image.

In patients receiving MSAP reconstruction (n=5), 30% of perforators intraoperatively used for flap elevation were visible in CE-mDIXON images. After the first two patients, we hypothesized that fat planes in THRIVE images could give an indication of perforator locations (**Figure 8**). Therefore, THRIVE images were evaluated in subsequent patients for potential perforators and were incorporated in the 3D model of patient 6. Taking both THRIVE and CE-mDIXON images into consideration, 50% of perforators used for flap elevation were incorporated in the 3D models. In patient 10, the intraoperatively identified perforators were small and not visible on MRA imaging nor incorporated in the 3D model. For perforators that were visible on MRA imaging, all perforators showed an intramuscular course that matched with intraoperative findings.

### 3.3 Case illustration with 3D-printed perforator guide

In patient 9, a patient-specific perforator guide was designed, 3D-printed and used for perforator marking as a first case to demonstrate the potential of our proposed workflow.

**Figure 9:**
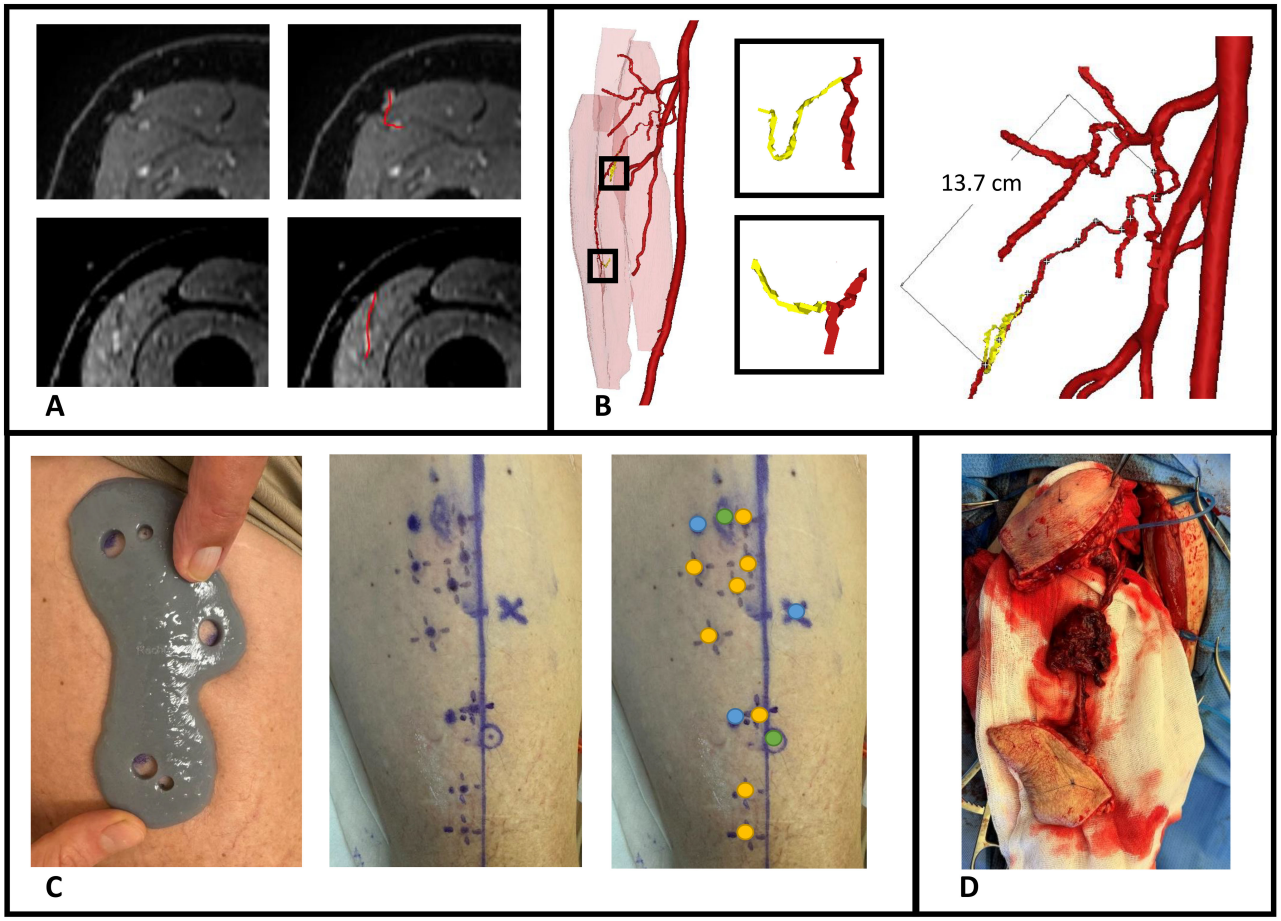
Case illustration of patient 9 receiving chimeric ALT flap using a 3D-printed perforator guide for perforator marking. **(A)** Axial slices of contrast-enhanced mDIXON images showing the perforators, **(B)** MRA-based 3D model displaying muscles in red, vessels in red, perforators in yellow and pedicle length measurement, **(C)** Marking of perforators with perforator guide and verification with handheld Doppler, **(D)** Elevated chimeric ALT flap with double skin paddle and muscle component. Blue dots, MRI markers; orange dots, positive arterial Doppler signal; green dots, marked perforators from 3D model with positive arterial Doppler signal.

This patient presented with a cutaneous melanoma of the cheek and required a chimeric soft tissue ALT flap with double skin paddle and muscle component. Preoperative MRA imaging and 3D modelling showed two suitable musculocutaneous perforators from the descending branch of lateral circumflex femoral artery (LCFA) with estimated pedicle length to the first perforator of 13.7 cm (**Figures 9a, b**). Before surgery, perforators were marked with the 3D-printed perforator guide and verified for a positive arterial signal with handheld Doppler (**Figure 9c**). In the operating room, extra Doppler measurements were performed to identify potential other perforators that were not identified in MRA images (**Figure 9c**). Intraoperatively, only the perforators from the MRA-based 3D model were found and dissected. A chimeric flap with two 9.5 x 6.0 cm skin paddles and a part of vastus lateralis muscle was successfully harvested and transplanted to the recipient site (**Figure 9d**). No postoperative complications occurred.

## 4 Discussion

The aim of the current study was to introduce and describe the workflow of a novel approach for perforator mapping that integrates contrast-enhanced Magnetic Resonance Angiography (MRA)-based 3D modelling with 3D-printing technology. This approach represents an important step toward virtual surgical planning and digital modelling in soft tissue reconstruction for head and neck cancer patients. By creating patient-specific 3D representations of vascular anatomy and translating them into customized, 3D-printed perforator guides, we offer a radiation-free and personalized solution. This technique allows to overcome shortcomings of current techniques, such as false positives in case of handheld Doppler, and is particularly advantageous in complex reconstructions involving chimeric flaps, multiple skin paddles, thinned flaps, or flaps requiring long vascular pedicles in vessel-depleted necks.

The usability questionnaire results indicate that surgeons perceive the proposed workflow as a valuable enhancement over the standard perforator mapping approach using handheld Doppler alone, with a median score of 5 out of 5. Additionally, the 3D models were found to improve interpretation of 2D MRA data (median score 4 out of 5), particularly in cases involving perforators with long intramuscular courses that required meticulous dissection. In several instances, surgeons noted that the 3D models clarified unexpected anatomical variations encountered intraoperatively. Compared to traditional perforator mapping techniques, such as handheld Doppler or 2D imaging, our 3D approach offers a comprehensive spatial overview of the vascular anatomy, facilitating more accurate perforator identification. This may reduce the risk of tissue and vascular injury during dissection, potentially leading to more efficient flap elevation and improved flap survival outcomes.

The pedicle lengths measured in the 3D models closely corresponded with intraoperative findings, showing a mean (SD) absolute difference of 1.0 (0.9) cm. In some cases, intraoperative measurements slightly exceeded preoperative estimates. For ALT and MSAP flaps, this discrepancy can be attributed to the curved trajectory of the pedicle on imaging, which becomes straightened during surgical dissection, resulting in a longer measured length intraoperatively. In FFF cases, intraoperative measurements are typically taken at the point where the perforator crosses the posterolateral septum, which is often distal to the peroneal origin used for measurement in the 3D model. Nevertheless, this difference is clinically insignificant in our opinion, as a longer vascular pedicle is generally advantageous in reconstructive procedures.

Our findings demonstrate that the CE-mDIXON sequence within our MRA protocol reliably visualizes the perforators selected intraoperatively for ALT and FFF flap elevation. In all cases, the inter- or intramuscular course of the perforators observed on MRA corresponded with intraoperative findings. When multiple suitable perforators are present, surgeons typically prefer larger perforators with an intermuscular course. Therefore, our workflow may support preoperative selection of the optimal perforator based on size, course, and pedicle length. Additionally, in cases where both legs are viable donor sites, the decision can be guided by the vascular anatomy and perforator distribution, enabling a more tailored surgical approach. Also, the preoperative insight may assist less experienced surgeons in navigating anatomical variability with greater confidence.

Although most perforators in FFF cases follow an intermuscular septal course - raising questions about the necessity of preoperative imaging in resource-limited settings - precise preoperative localization of these vessels offers substantial advantages during virtual surgical planning. In particular, when designing bony reconstructions and surgical cutting guides, the integration of detailed vascular anatomy - including 3D pedicle length, perforator origin, and fascial exit points - facilitates safer dissection and enhances the predictability of flap harvest. This level of planning enables optimal alignment of bone segments with desired skin paddles, which is especially critical in complex reconstructions, such as multi-segment defects or through-and-through defects requiring both intraoral and extraoral skin components. In such scenarios, more proximal perforators may be required to accommodate specific reconstructive configurations. Notably, we have observed that these proximal perforators more frequently exhibit an intramuscular course through the soleus muscle, in contrast to the predominantly intermuscular septal trajectory of distal perforators. Furthermore, the inclusion of soleus perforating branches may also allow for the incorporation of a muscle component as part of a chimeric flap, offering additional reconstructive versatility in complex oromandibular defects (29–31).

For MSAP flaps, 70% of the intraoperatively identified perforators were not visible on CE-mDIXON images, and when visible, they appeared less distinct compared to those in ALT and FFF cases. This is likely due to the smaller caliber of MSAP perforators, which may fall below the resolution threshold of the current imaging protocol. However, since MSAP perforators typically course through fat within the medial gastrocnemius muscle, these fat planes can be visualized on THRIVE images. In the five MSAP cases included, we retrospectively reviewed axial THRIVE slices for fat planes traversing the gastrocnemius and penetrating the deep fascia towards the skin. In four cases, these fat planes corresponded with intraoperative findings of perforator locations but more patients should be evaluated in future research to confirm our preliminary results. Although MRA and 3D modelling did not consistently visualize the perforators themselves, they did clearly depict the medial sural artery (MSA) branching pattern, which surgeons found valuable for selecting a MSA sub-branch for flap elevation. Based on these findings, we recommend a multi-parametric MRA-based 3D modelling approach: using CE-mDIXON for MSA branching and THRIVE for identifying potential perforator-associated fat planes. Real-time imaging modalities, such as high-frequency color Doppler ultrasonography, may further aid in confirming perforator presence within these fat planes (32, 33).

Our findings are consistent with previous studies that have demonstrated the applicability of contrast-enhanced MRA imaging for perforator mapping in FFF (14, 34–39) and ALT flaps (21, 40, 41). These studies support MRA as a reliable, non-invasive, radiation-free alternative to CTA, offering superior vessel-to-muscle and muscle-to-muscle contrast that is particularly relevant for the identification of intramuscular perforating branches. Notably, to date, MRA has not been described in the literature as method for perforator mapping in MSAP flaps. Furthermore, no prior studies have combined contrast-enhanced MRA with 3D modelling and 3D-printed guides for perioperative perforator localization, highlighting the novelty of our approach.

The use of vascular 3D modelling and 3D-printed perforator guides has been reported in a limited number of studies involving CTA-based workflows. Battaglia et al. (42) and Wei et al. (43) demonstrated the feasibility of 3D-printed guides for perforator localization in FFF planning, while Li et al. (44) used a similar technique for ALT flaps. Our study is the first to apply this concept using MRA data, offering a radiation-free and nephrotoxicity-free alternative that is particularly relevant for patients undergoing extensive oncologic treatment. Moreover, MRI holds the potential for non-contrast-enhanced vascular imaging, which could eliminate the need for intravenous contrast (45, 46).

Although our approach seems promising, several limitations must be acknowledged. First, the workflow depends on access to high-resolution MRA imaging and a robust CAD-CAM infrastructure, which may not be readily available in all clinical settings. Compared to CTA, MRA is associated with higher costs and longer acquisition times. Although 3D-printing technology is becoming more affordable and accessible, it may still pose a barrier to widespread implementation. Second, the validation of the 3D models was conducted in a retrospective manner and the clinical accuracy of the 3D-printed perforator guides in delineating the perforator locations on the patient’s skin has yet to be prospectively validated. Third, in our evaluated cases, handheld Doppler was used as a reference standard despite its known limitations and variable accuracy. Nevertheless, we chose to include Doppler in our analysis, because it remains the most commonly used and widely accessible tool for perforator localization in routine clinical practice. Additional to Doppler, intraoperative confirmation of the presence and course of the perforators was recorded and used as the ground truth, as it remains the most reliable and clinically relevant method for validation. Lastly, the relatively small sample size limits generalizability of our findings. To address these limitations, a larger prospective clinical study involving FFF and ALT patients is currently underway. This study aims to evaluate the reliability of preoperative virtual surgical flap planning, the delineation accuracy of the 3D-printed perforator guides, and the overall impact of the digital workflow on surgical outcomes such as flap harvest time and flap survival.

Despite its limitations, this study offers several key contributions to the field of reconstructive surgery. To our knowledge, this is the first study to integrate MRA imaging with 3D modelling and 3D-printing for patient-specific perforator mapping in head and neck reconstruction. Importantly, the proposed workflow is adaptable and can be extended to other perforator flaps, such as the deep inferior epigastric artery perforator (DIEP) flap and the superficial circumflex iliac artery perforator (SCIP) flap. Additionally, the workflow can be modified for use with CTA in settings where MRA is not available.

In conclusion, preoperative contrast-enhanced MRA imaging represents a promising, radiation-free, and nephrotoxicity-free alternative to CTA for perforator mapping in head and neck reconstructive surgery. The integration of 3D modelling and 3D-printing provides a practical and intuitive method for translating imaging data into the operating room, potentially enabling more efficient, patient-tailored flap design and elevation, and improved surgical outcomes. Future research should focus on validating this workflow in a larger, prospective clinical study involving FFF and ALT patients, with particular attention to the delineation accuracy of the 3D-printed guides, added value for surgeons, and impact on surgical outcomes. Additionally, further optimization of the MRA protocol may enhance the visualization of smaller perforators, such as those in MSAP flaps. In this context, the administration of sublingual nitroglycerin as a vasodilator could be investigated in future studies to assess its potential to enhance the detectability of small-caliber perforators (47, 48). Ultimately, combining this perforator mapping approach with defect modelling at the tumor site could pave the way for a fully digital, virtual surgical planning workflow, enabling personalized, precise, and reproducible head and neck reconstructive surgery.

## Data Availability

The datasets presented in this article are not readily available because of the patient data that cannot be shared outside our organisation. Requests to access the datasets should be directed to a.d.geer@nki.nl.
